# Characteristics of pediatric-onset anti-contactin1 antibody-associated autoimmune nodopathies with concomitant membranous nephropathy

**DOI:** 10.1186/s12887-025-06429-3

**Published:** 2026-01-16

**Authors:** Qiuyue Guan, Yi Xie, Yonghua He, Liru Qiu, Yan Liu

**Affiliations:** Department of Pediatrics, Tongji Hospital, Tongji Medical College, Huazhong University of Science and Technology, Wuhan, 430030 PR China

**Keywords:** Contactin-1, Autoimmune nodopathy, Membranous nephropathy, Pediatric

## Abstract

**Background:**

Anti-contactin-1 antibody-associated autoimmune nodopathy (CNTN1-AN) is a rare disorder predominantly affecting older individuals, characterized by sensorimotor peripheral neuropathy, with over 50% of cases presenting with proteinuria and membranous nephropathy (MN). Pediatric-onset CNTN1-AN is exceptionally rare, and its clinical profile remains poorly characterized.

**Case presentation and literature review:**

We report a pediatric case of CNTN1-AN with MN and conduct a literature review to elucidate the pathogenesis and clinical features of CNTN1-AN combined with MN in children. The patient, an 11-year-old girl, presented with a one-month history of progressive lower limb weakness and a 10-day progression of gait instability. Physical examination demonstrated bilateral proximal lower limb weakness, restricted mobility, and sensory abnormalities.No facial or limb edema or frothy urine was observed. Elevated anti-CNTN1 antibody titers were detected in both serum (1:320) and cerebrospinal fluid (CSF) (1:3.2). Urinalysis showed significant proteinuria (4+). Electromyography (EMG) indicated peripheral nerve involvement, and lumbosacral and brachial plexus magnetic resonance imaging (MRI) demonstrated nerve root edema and thickening. Renal biopsy confirmed stage I MN, establishing the diagnosis of CNTN1-AN with MN. Following prednisone and rituximab therapy, motor function improved, though renal progression persisted. A comprehensive literature review identified six confirmed pediatric CNTN1-AN cases globally. Including the current case, renal involvement was observed in 3/7 pediatric cases, with MN confirmed in two. Comparative analysis indicates that CNTN1-AN manifests similarly across pediatric and adult populations, though pediatric cases demonstrate potentially lower incidence rates.

**Conclusion:**

Pediatric CNTN1-AN exhibits a low incidence, requiring ongoing renal function monitoring in affected cases. In CNTN1-AN with MN, neurological symptoms respond well to low-dose rituximab, whereas renal manifestations often require intensified regimens. The mechanisms linking CNTN1-AN and MN remain elusive, highlighting the need for further studies to optimize rituximab therapeutic protocols.

**Graphical Abstract:**

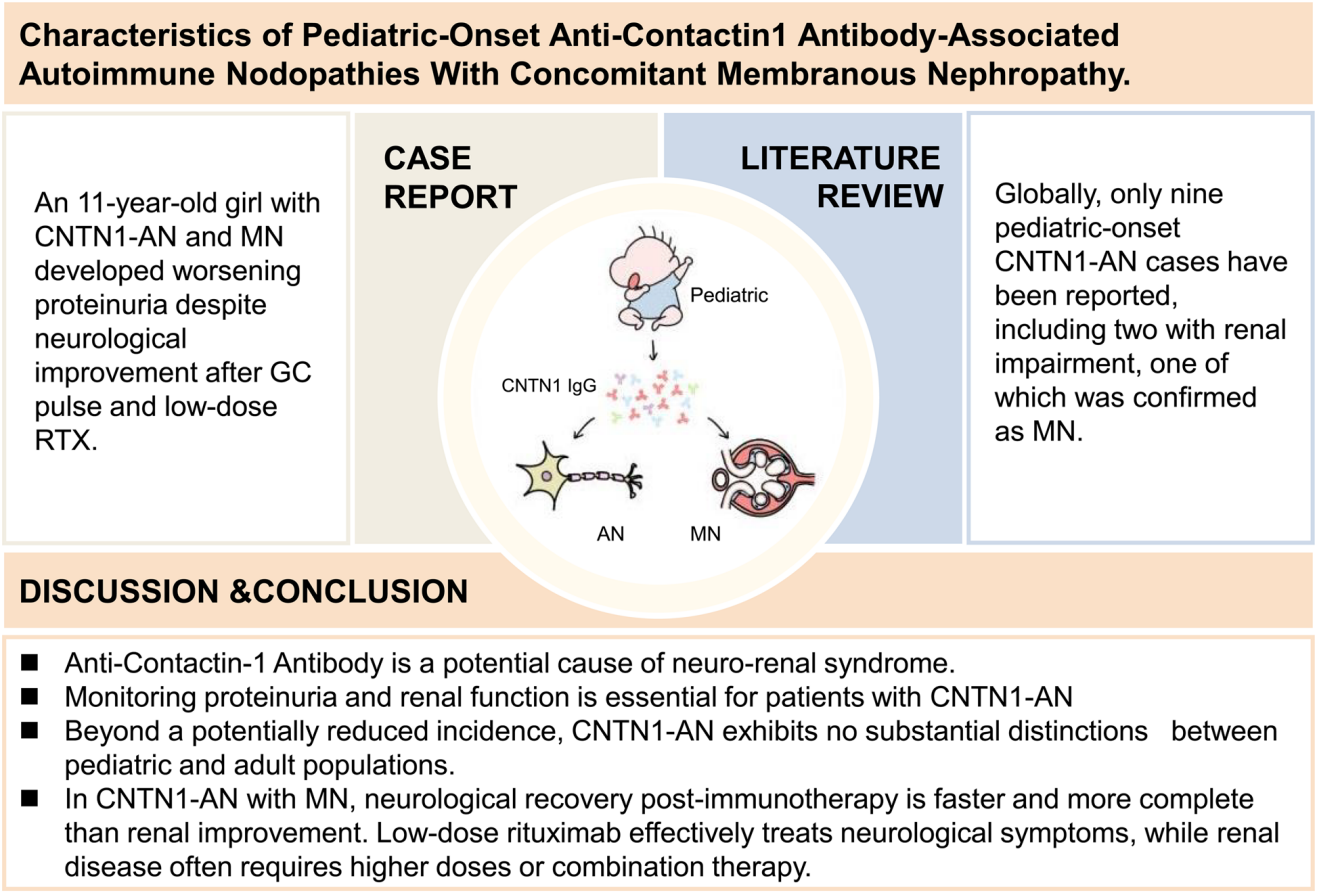

## Introduction

Emerging evidence reveals distinct clinical characteristics in patients with autoantibodies against nodal/paranodal proteins (NF186, NF155, CNTN1, Caspr1), showing significantly different pathological, electrophysiological, and therapeutic patterns distinct from classic chronic inflammatory demyelinating polyradiculoneuropathy (CIDP). According to the 2021 European Academy of Neurology/Peripheral Nerve Society (EAN/PNS) classification, CIDP patients with anti-nodal antibodies are now categorized under the newly defined entity of AN [1]. AN is characterized by acute, subacute, or chronic sensorimotor peripheral neuropathy and requires careful differentiation from Guillain-Barré syndrome (GBS) and CIDP.

The CNTN1/Caspr1 complex, localized at the axolemma of the paranodal region of the node of Ranvier, interacts with NF 155 at the myelin terminus to form an axo-glial septate-like junction. This junction stabilizes the myelin sheath attachment to the axolemma and maintains the spatial segregation of voltage-gated Na⁺ channels in the nodal region from voltage-gated K⁺ channels in the juxtaparanodal region. However, anti-CNTN1 IgG4 antibodies can infiltrate the paranodal region, disrupting the interaction between CNTN1 and other paranodal proteins. This disruption causes detachment of the myelin terminal loops from the juxtaparanodal axolemma, nodal widening, incr eased nodal capacitance, and current leakage. Consequently, reduced forward current and ectopic juxtaparanodal hyperpolarization occur due to the mislocalization of voltage-gated K⁺ channels. These pathological changes lead to slowed nerve conduction velocity, conduction block, and ultimately manifest as limb weakness and sensory deficits.

MN is an autoimmune disorder characterized by the subepithelial deposition of immune complexes, consisting of immunoglobulin G (IgG) and target antigens, along the glomerular basement membrane (GBM). This condition is primarily driven by immune system activation triggered by various podocyte membrane antigens.In 2021, Le Quintrec et al. [2] identified CNTN1 as a novel target antigen in CIDP-associated MN. Subsequent studies have reported a rising incidence of CNTN1-AN cases with concurrent MN, particularly among middle-aged and elderly male populations. In contrast, pediatric-onset CNTN1-AN with MN remains exceptionally rare, with limited documented cases, and its clinical characteristics remain to be explored.

## Case presentation and literature review

### Case presentation

#### Medical history and physical examination

An 11-year-old girl was admitted to Pediatric Neurology in September 2024, with progressive bilateral lower limb weakness over one month and gait instability for 10 days. Her medical, developmental, and family histories were unremarkable. Physical examination revealed no evident skin rash, no joint swelling or tenderness, and no oral ulcers. Frothy urine was not observed. Neurological examination revealed grade 5 strength in the distal upper and lower limbs, but only grade 3 strength in the proximal lower limbs. Romberg’s sign was negative, and finger-to-nose testing was stable. However, she could not walk in a straight line. Sensory testing showed decreased tactile, pain, and complex pattern sensation in the feet and lower legs. Her gait was abnormal with reduced foot lift, and she was unable to squat, jump, or climb stairs. She reported lumbago and foot numbness after walking.

#### Auxiliary examination

Routine laboratory investigations, including complete blood count, renal function tests, electrolyte panel, coagulation profile, blood ammonia, lactate, glucose, pyruvate, anti-phospholipase A2 receptor antibodies, folic acid, vitamin B12, complement levels (C3 0.3 g/L, reference 0.1–0.4 g/L; C4 1.48 g/L, reference 0.8–1.6 g/L), anti-dsDNA, and anti-Smith antibodies were all within normal ranges. Peripheral blood testing revealed a positive antinuclear antibody (ANA) titer of 1:100 (granular pattern), and an elevated erythrocyte sedimentation rate of 35 mm/h (reference range: 0–20). Serum analysis showed reduced immunoglobulin G (5.45 g/L; reference range: 8–16), hypoalbuminemia (31.9 g/L; reference range: 40–55), and hypoproteinemia (56 g/L; reference range: 66–87), along with hypercholesterolemia (6.56 mmol/L; reference range: <5.18), hyperuricemia (430 µmol/L; reference range: 142.8–339.2), and vitamin D deficiency (25-hydroxyvitamin D: 8.4 ng/mL). Urinalysis demonstrated hematuria(1+), significant proteinuria (4+), and an elevated urine albumin-to-creatinine ratio of 1445.1 µg/mg (reference range: <30). Urinary N-acetyl-β-D-glucosaminidase was elevated at 19.5 U/L (reference range: 0.3–11.5), with 24-hour urine total protein 2196.8 mg/L.

**Fig. 1:**
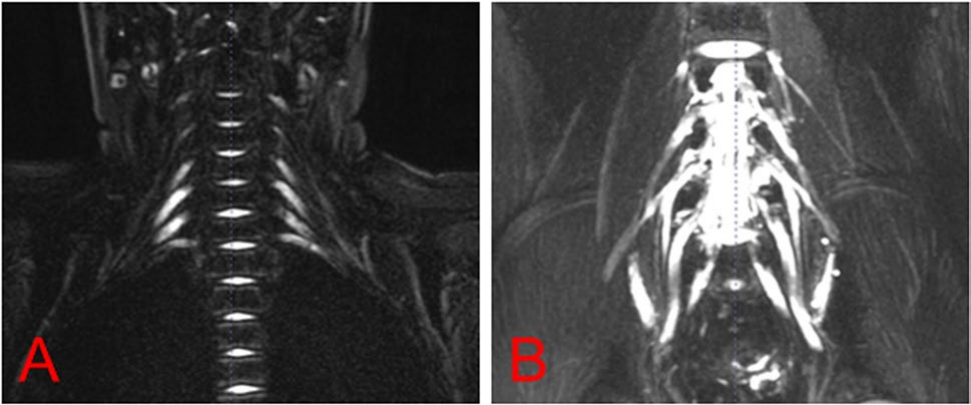
Neuroimaging. (**A**) MRI showed thickened bilateral brachial plexus nerves with T2 hyperintensity, without distortion or disruption. (**B**) revealed linear and patchy T1 and T2 hyperintensities in L1-L3 subcutaneous fat, with mild thickening and T2 hyperintensity in bilateral L4-5 nerve roots

Peripheral neuropathy antibody testing revealed elevated anti-CNTN1 IgG titers (serum: 1:320; CSF: 1:3.2) via cell-based assay (CBA), while other serum and CSF peripheral neuropathy antibodies were negative, including GQ1b, GM1, myelin-associated glycoprotein (MAG), sulfatide, NF155, NF186, and CASPR1. CSF analysis showed a nucleated cell count of 5 × 10^6/L, elevated total protein (2266 mg/L), albumin (1440 mg/L), and immunoglobulins (IgG: 169.0 mg/L; IgM: 11.4 mg/L; IgA: 44.1 mg/L). EMG indicated peripheral nerve involvement in both upper limbs and the right lower limb, affecting motor, sensory, distal, and proximal nerve roots, with evidence of both demyelinating and axonal damage, predominantly demyelinating motor fiber involvement (Table 1). MRI of the lumbosacral and brachial plexus revealed bilateral L4-5 nerve root swelling and thickening, and inflammatory changes in the bilateral brachial plexus (Fig. 1).

**Table 1:** Nerve conduction studies (NCS) results

	Normal	Case presentation	Case presentation after treatment
Times from onset		1 month	3.5 months
Side		L/R	L/R
Median nerve			
DL (ms)	≤4.2	Wrist 6.25/6.75	Wrist 7.2/7.2
		Elbow 10.75/10.85	Elbow 12.1/11.3
MCV (m/s)	≥50.0	45.8/48.3	44.8/51.2
CMAP amplitude (mV)	≥4.8	Wrist 7.67/9.78	Wrist 9.58/11.81
		Elbow 6.11/9.11	Elbow 8.13/11.28
F wave latency (ms)	≤30.0	44.75/43.15	45.1/41.9
F wave response frequency	≥79%	100%/0%	100%/100%
SCV (m/s)	≥50.8	39.0/41.2	32.0/33.9
SNAP amplitude (µV)	≥8.0	24.9/23.9	28.05/22.63
Ulnar nerve			
DL(ms)	≤3.1	Wrist 3.80/4.85	Wrist 5.0/4.0
		Elbow 11.35/13.80	Elbow 11.1/9.7
MCV (m/s)	≥51.0	31.9/25.1	31.1/33.3
CMAP amplitude (mV)	≥5.5	Wrist 3.16/2.68	Wrist 4.06/3.01
		Elbow 0.39/1.17	Elbow 3.32/3.12
F wave latency (ms)	≤30.0	46.35/44.55	Not examined
F wave response frequency	≥79%	29%/0%	Not examined
SCV (m/s)	≥50.6	46.2/59.3	36.2/45.4
SNAP amplitude (µV)	≥5.0	13.6/22.8	11.27/18.08
Tibial nerve			
DL(ms)	≤5.8	R Ankle 4.85	Ankle 7.4/6.6
		R Popliteal 13.15	Popliteal 16.3/15.4
MCV (m/s)	≥39.4	R 41.6	39.3/39.7
CMAP amplitude (mV)	≥5.0	R Ankle 4.89	Ankle 2.34/2.09
		R Popliteal 1.30	Popliteal 1.45/1.92
F wave latency (ms)	≤60.0	83.9/79.6	73.2/71.4
F wave response frequency	≥79%	0%/0%	56.3%/100%
Peroneal			
DL (ms)	≤4.6	R Ankle 5.95	Ankle 7.4/7.7
		R Head of fibula 13.15	Head of fibula 14.7/15.2
MCV (m/s)	≥39.8	R 40.7	36.9/38.6
CMAP amplitude (mV)	≥2.3	R Ankle 1.58	Ankle 0.49/1.13
		R Head of fibula 1.28	Head of fibula 0.35/0.65
Superficial peroneal			
SCV (m/s)	≥41.3	R Not elicited	48.0/47.3
SNAP amplitude (µV)	≥4.0	R Not elicited	10.88/6.99
Sural			
SCV (m/s)	≥41.9	R Not elicited	50.0/50.0
SNAP amplitude (uV)	≥7.0	R Not elicited	8.75/7.67

*μV* microvolt, *mV* millivolt, *m/s* meter per second, *ms* millisecond, *CMAP* compound muscle action potential, *DL* distal motor latency, *MCV* motor nerve conduction velocity

**Fig. 2:**
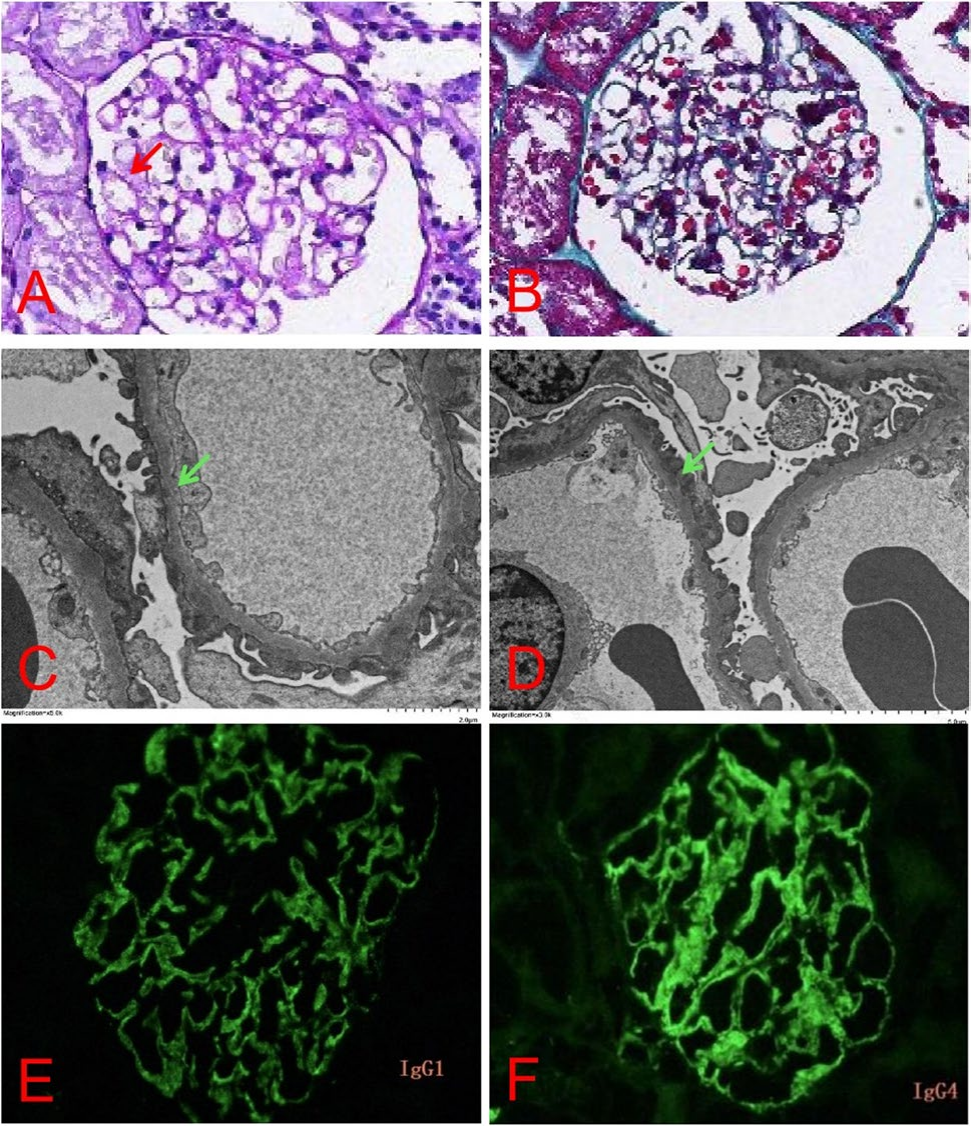
Pathological results of renal biopsy. Light microscopy of 30 glomeruli revealed 2 with global sclerosis and 2 with segmental sclerosis. Focal mild mesangial hypercellularity and matrix expansion were noted. The glomerular basement membrane showed ribbon-like remodeling with early spike formation (PAS stain, red arrow). Tubulointerstitial changes included focal tubular epithelial degeneration and patchy interstitial fibrosis (**A** and **B**). Electron microscopy revealed diffuse podocyte foot process effacement with microvillous transformation, scattered subepithelial electron-dense deposits (green arrow), and segmental mild mesangial hypercellularity and matrix expansion (**C** and **D**). Renal tissue cryosection immunofluorescence showed CNTN1 antibody IgG1++(**E**),IgG2+,IgG3+,IgG4++/+++(**F**),with granular deposits in glomerular capillary loops

Renal biopsy confirmed MN (stage I) on light microscopy. Immunofluorescence staining of frozen renal tissue demonstrated granular deposition of CNTN1 antibody IgG1 (++), IgG2 (+), IgG3 (+), and IgG4 (++/+++) along glomerular capillary loops. Paraffin immunofluorescence staining was negative for phospholipase A2 receptor (PLA2R), neural epidermal growth factor-like 1 (NELL-1), thrombospondin type 1 domain-containing 7 A (THSD7A) and etc. (Fig. 2). Additional imaging studies, including renal ultrasound, electrocardiogram, cranial MRI, and thoracic spine MRI, revealed no significant abnormalities.

#### Diagnosis and treatment

**Fig. 3:**
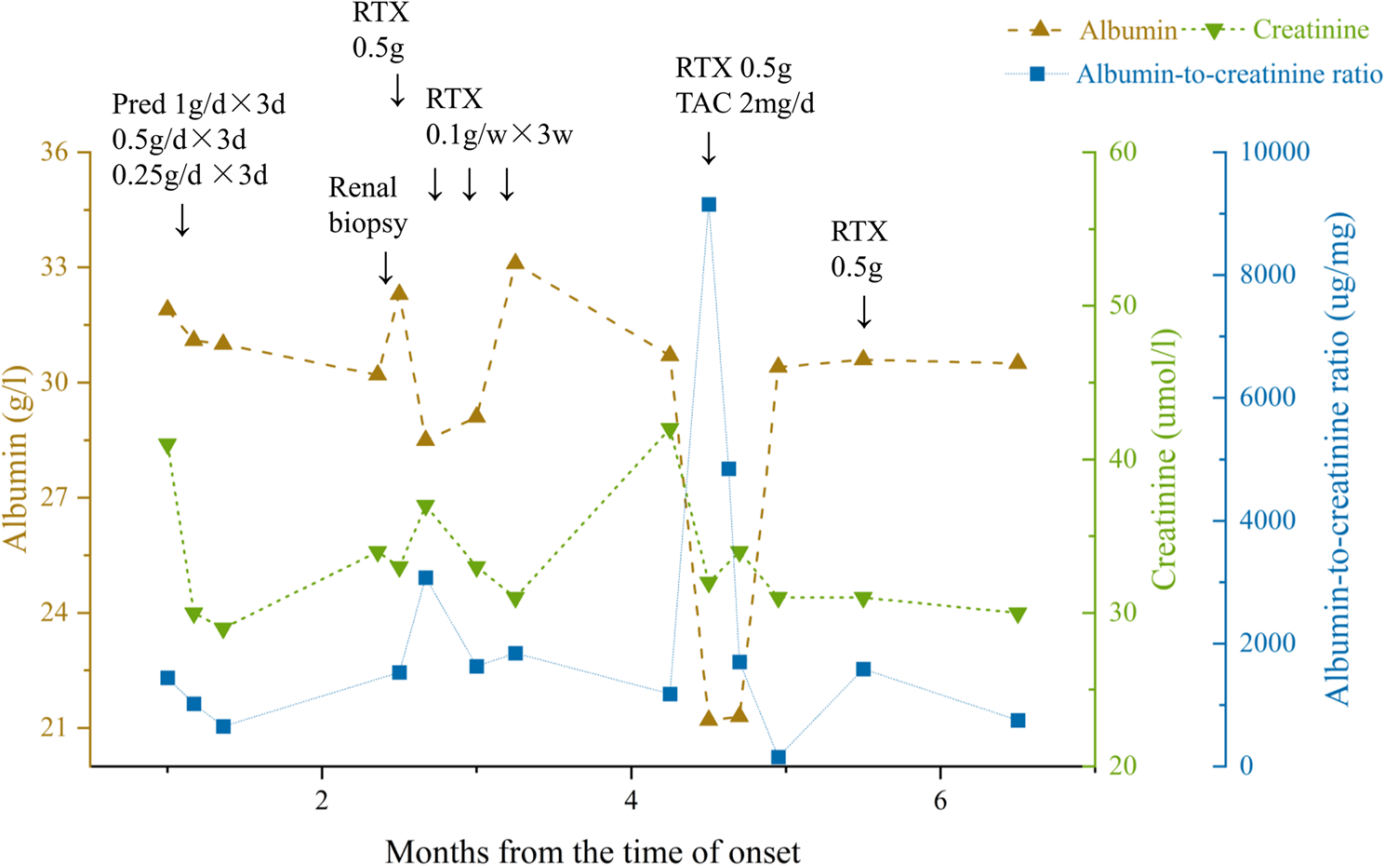
The clinical course of the current case. RTX: rituximab; Pred: prednisone; TAC: tacrolimus

Based on the above symptoms, signs, and diagnostic findings, the patient was diagnosed with CNTN1-AN. The patient underwent prednisone pulse therapy followed by oral prednisone for one month. Subsequently, she achieved independent stair climbing and improved tactile, pain, and complex sensory function in the lower limbs; however, proximal lower limb strength did not further improve. Persistent proteinuria (± to +++) prompted a renal biopsy, confirming concurrent MN. Given the predominant IgG4 pathology of CNTN1-AN and the documented poor response to intravenous immunoglobulin (IVIG) in such cases, we opted for B-cell depletion therapy with rituximab as the primary immunomodulatory strategy [3–5]. She then received rituximab therapy (initial dose:500 mg, followed by weekly doses of 100 mg). After four rituximab doses, she could independently squat and stand, with proximal muscle strength improving to grade 4 + in all four limbs. Sensation in the calves and feet normalized. Mild gait instability continued, with inability to walk in a straight line. At 3.5 months into the disease course, urine protein remained 2+, serum anti-CNTN1 IgG titers persisted at 1:320, and EMG revealed ongoing peripheral nerve involvement in both upper and lower limbs, affecting motor axons, myelin, and nerve roots (Table 1). At 4.5 months, the patient developed limb edema. A 7-day trial of irbesartan (2 mg/kg/day) was discontinued due to dizziness, hypotension, and urticarial rash. Follow-up tests showed urine protein (4+), elevated urine albumin-to-creatinine ratio (9156.2 µg/mg), decreased albumin (21.1 g/L), and creatinine (32 µmol/L). Immunoglobulin G (IgG) was reduced to 2.5 g/L, and complement C3 was elevated to 1.59 g/L. A 6-minute walk test showed a distance of 395 m. Given the patient’s demonstrated hypersensitivity to irbesartan with concern for potential cross-reactive adverse effects, along with the presence of severe, active nephrotic syndrome requiring more definitive intervention, we decided not to trial alternative RAASi and instead, in accordance with the KDIGO 2021 Glomerular Disease Guidelines [6], oral tacrolimus (2 mg/day) was initiated, with trough concentrations fluctuating between 4.01 and 7.84 ng/ml. Additionally, the dose of rituximab was increased. The patient received a fifth rituximab pulse (500 mg), followed by a sixth rituximab pulse (500 mg) one month later. Follow-up tests at 6.5 months showed reduced urine protein (2+) and a decreased urine albumin-to-creatinine ratio (752.9 µg/mg). Albumin was 30.5 g/L (Fig. 3). There was significant improvement in limb mobility, with proximal muscle strength graded at 5-. To monitor the biological response to rituximab, B-cell (CD19+) counts were serially assessed. The pre-treatment B-cell count was 743 cells/µL. Following rituximab administration, complete B-cell depletion (0 cells/µL) was achieved and sustained on subsequent measurements, confirming effective pharmacological targeting.

### Literature review

Literature was identified from PubMed database, using the search terms “CIDP,” “anti-CNTN1,” “autoimmune neuropathy,” “MN,” and “membranous glomerulopathy.” The search encompassed studies and case reports published up to November 2024. After removing duplicates and merging relevant records, we identified over 100 documented cases of CNTN1-AN [3, 7–14]. Among these, only 9 cases involved patients with an onset age under 18 years. Among the 9 cases, 3 lacked detailed clinical descriptions. Of the remaining 6 cases with complete clinical data, only 1 was associated with concurrent MN (Table 2).

**Table 2:** Clinical features of pediatric CNTN1-AN and CNTN1-AN with MN

	Patient 1	Patient 2, 3	Patient 4	Patient 5	Patient 6	Patient 7
Year/author	2019/Carrera-García L et al. [10]	2020/De Simoni et al. [11]	2023/Merel C Broers et al. [12]	2024/Chen J et al. [13]	2023/Tang Y et al. [14]	2025/ this article
Age of onset(years)/sex	2.75/M	7.9(mean age)/NA	Toddler/F	12/M	14/M	11/F
Age of diagnosis(years)	7.75	NA	NA	20	NA	11
Mode of onset/ course of disease	Actue, relapsing	Chronic	Actue	Chronic	Actue, relapsing	Actue
Preceding infection	-	NA	NA	-	+	+
Initial symptoms	Lower limb weakness and frequent falls	NA	Bilateral lower limb weakness and pain, complete dependence on a wheelchair	Limb tremors, slurred speech, and mild limb weakness	Lower limb weakness and numbness	Progressive weakness in the lower limbs
Sequence of manifestations	No renal involvement	NA	NA	AN→Renal involvement, 8 years	Concurrent	Concurrent
Tremble	-	+/+	+	+	-	-
Ataxia	+	+/+	NA	+	+	+
Weakness	LD > LP > UP = UD	NA	L > U	L > U	LP > LD > UP = UD	LP > LD > UP = UD
Numbness	-	NA	NA	+	+	-
Pain	-	+/+	+	-	+	+
Autonomic symptoms	Diarrhea	NA	-	Orthostatic dizziness	+	Hyperhidrosis
Dysarthria	-	NA	NA	+	-	-
Cranial nerve damage	-	One case of optic neuritis	-	-	-	-
Nystagmus	-	NA	NA	+	-	-
Sensory disturbances	-	NA	NA	Distal lower limb pain with impaired vibration sense.	Hyperalgesia with impaired vibration sense.	Impaired touch, pain, and vibration sense in bilateral calves and feet.
Muscle atrophy	-	NA	NA	+	-	-
CNTN1 antibody	+	+	Serum 1:6400, IgG1 ±, IgG2 ±, IgG3 -, and IgG4 +	Serum 1:100,CSF 1:10	+	Serum 1:320,CSF 1:3.2
Autoantibodies	NA	NA	NA	ANA 1:200	NA	ANA 1:100
Serum anti-PLA2R antibodies	NA	NA	NA	NA	-	-
24 h-UTP (g/24 h)	NA	NA	NA	5.1	5.46	2.2
Serum creatinine(µmol/l)	NA	NA	NA	NA	42	41
CSF protein(g/l)	1.48	2.924(mean value)	Elevated CSF protein	3.009	2.84	2.266
CSF cells(*10^6/L)	Normal	4.6(mean value)	NA	12	1	5
Protein-cell dissociation	+	+/+	NA	+	+	+
Neurological MRI	NA	NA	NA	Diffuse thickening of bilateral cervical, lumbosacral nerve roots and cauda equina, with multiple intercostal root sheath cysts.	Lumbosacral nerve MRI was normal	Bilateral L4-5 nerve root hypertrophy with brachial plexus inflammatory changes.
Electrophysiological studies	Prolonged distal latencies, slowed conduction velocities, reduced CMAP amplitudes, and chronic denervation with fibrillations and positive sharp waves in multiple motor nerves.	NA	Consistent with EFNS/PNS 2010 diagnostic criteria	Severe demyelination and axonal injury	Multiple motor conduction blocks without temporal dispersion, prolonged distal latencies, slowed velocities, and reduced CMAP amplitudes (lower limb predominance).	Demyelination and axonal damage were observed in the peripheral nerves of both upper limbs and the right lower limb, with predominant demyelination of motor fibers
Nerve biopsy	NA	NA	NA	NA	Demyelination combined with axonal damage	NA
Renal biopsy	NA	NA	NA	NA	MN (stage I)	MN (stage I)
Treatment	IVIG、GC	IVIG、GC、RTX	IVIG、GC	IVIG、GC、PLEX 、RTX	GC	GC、RTX、TAC
Prognosis	Initial IVIG (2 g/kg) yielded transient improvement, followed by clinical progression confirmed by CSF and EMG. A second IVIG course was ineffective, but GC (30 mg/kg/day × 5 days) achieved significant response. Disease relapsed at 1 year but responded to IVIG retreatment.	Patient 2 worsened after 4 months of IVIG + GC but rapidly improved post-RTX. Patient 3 showed partial IVIG response but relapsed post-infection; RTX induced significant improvement, achieving mRS 2 at final follow-up.	Following IVIG failure, Patient 4 achieved GBS disability score 2 with RTX + GC, ambulating with braces.	Patient 5 showed progressive deterioration despite 9-year intermittent IVIG + GC therapy. Three PLEX sessions were ineffective, but single-dose RTX (500 mg) induced marked clinical improvement.	NA	GC and low-dose RTX improved motor function but not renal progression. RTX dose escalation significantly reduced proteinuria.

*F* female, *M* male, *NA* unknown, *LP* lower proximal, *LD* lower distal, *UP* upper proximal, *UD* upper distal, *UTP* urinary total protein, *mRS* modified Rankin Scale, *ANA* antinuclear antibody, *IVIG* Intravenous Immunoglobulin, *GC* Glucocorticoids, *RTX* rituximab, *TAC* tacrolimus, *PLEX* Plasma Exchange

## Discussion

### Comparative clinical characteristics between pediatric and adult populations with CNTN1-AN

Patients with CNTN1-AN typically present with an older age of onset, a male predominance, and acute or subacute onset, often accompanied by a preceding infection [14]. Some cases may initially be misdiagnosed as acute GBS [3, 4]. The clinical manifestations primarily include sensory ataxia, symmetric limb weakness, paresthesia, neuropathic pain, tremors, numbness, and autonomic dysfunction. Some patients may also exhibit involvement of the respiratory system and cranial nerves(predominantly manifesting as facial palsy, ophthalmoplegia, and diplopia) [13, 15–17]. Over half of the patients exhibit proteinuria, renal involvement, and MN, with renal biopsy revealing deposition of IgG4 along the GBM [3, 4, 18–20]. The diverse clinical spectrum of CNTN1-AN is linked to the expression pattern of CNTN1, which is detectable in the retina, spinal cord, cerebral cortex, hippocampus, cerebellum, and glomeruli. However, it remains to be definitively confirmed whether CNTN1 is expressed by podocytes [5, 21]. Sensory ataxia is associated with the widespread expression of CNTN1 in the dorsal root ganglia of sensory nerves, where CNTN1 antibodies can cross the relatively permeable blood-nerve barrier. CNTN1 antibodies may disrupt CNTN1-PTPα interactions, inhibiting Fyn and Src kinase activity and contributing to cerebellar dysfunction [13].

Nerve biopsy findings typically show nodal elongation, paranodal disorganization, and myelin loop detachment. Notably, characteristic pathological features of classic CIDP are absent, such as segmental demyelination and onion-bulb formations resulting from Schwann cell proliferation [22]. EMG reveal prolonged distal motor latency (DML), reduced nerve conduction velocity (NCV), and the presence of conduction blocks without waveform dispersion. F-wave latency is either prolonged or absent, compound muscle action potential (CMAP) amplitude is significantly reduced, sensory conduction velocity (SCV) is slowed, and sensory nerve action potential (SNAP) amplitude is diminished [23]. EMG findings in this pediatric case align with those reported in previous studies, including prolonged DML and reduced CMAP amplitude in the median and ulnar nerves of both upper limbs, as well as markedly prolonged F-wave latency and decreased occurrence rates in the median, ulnar, and tibial nerves bilaterally.

Patients with CNTN1-AN demonstrate significantly elevated CSF protein levels, accompanied by a distinct protein-cell dissociation. To date, there is no evidence supporting intrathecal synthesis of CNTN1 antibodies. Neuroimaging findings in CNTN1-AN patients commonly show contrast enhancement or hypertrophy of nerve roots in both lumbosacral and brachial plexus regions [24]. Experimental studies have shown that CNTN1 antibodies bind to hippocampal neurons and cerebellar tissue in rats. Some researchers hypothesize that the elevated CSF protein levels may result from the relatively permeable blood-nerve barrier at the nerve roots, facilitating the infiltration of peripheral circulating proteins into the central nervous system [16].

Reports on pediatric CNTN1-AN remain limited in the literature. In 2019, Carrera-García et al. [10] reported the first pediatric case of CNTN1 antibody-associated CIDP. Subsequently, in 2020,De Simoni D et al. [11] conducted a CBA screening for CNTN1 antibodies in 54 children diagnosed with GBS or CIDP, identifying two CIDP cases positive for CNTN1 antibodies. A 2024 study cohort of 31 CNTN1-AN patients revealed that 3 (9.35%) were pediatric cases, and 11 (35.5%) had concurrent renal involvement, including 5 (16.1%) diagnosed with membranous glomerulonephritis [3].

Through a literature review, we identified over 100 reported cases of CNTN1-AN, with only 9 exhibiting pediatric onset, suggesting a higher incidence in adults. We retrospectively analyzed six pediatric CNTN1-AN cases with complete data, supplemented by the case presented in this report (Table 2). The findings revealed that limb weakness was the most common initial symptom (five cases, 71.4%), with a subset of patients reporting preceding infections (two cases, 28.5%). Renal involvement was observed in three cases (Patients 5–7), of which 66.7% were diagnosed with stage I membranous nephropathy. Neurological and renal symptoms frequently occurred concurrently. The most prevalent clinical manifestations included: ataxia (six cases, 85.7%); limb weakness (five cases, 71.4%), predominantly affecting the lower limbs and proximal muscles; pain (five cases, 71.4%); tremor (four cases, 57.1%); autonomic symptoms (four cases, 57.1%), such as hyperhidrosis and diarrhea; and sensory disturbances, primarily involving vibration and pain sensation (three cases, 42.9%). Additionally, some patients exhibited symptoms including numbness, dysarthria, cranial nerve involvement (e.g., optic neuritis), and muscle atrophy. CSF analysis in six pediatric patients demonstrated protein-cell dissociation. NCS indicated more significant involvement of motor fibers compared to sensory fibers. In the early disease stages, partial responsiveness to intravenous immunoglobulin (IVIG) and glucocorticoids (GC) was observed; however, rituximab demonstrated superior efficacy overall. In summary, beyond a potentially lower incidence, CNTN1-AN shows no substantial differences between pediatric and adult populations.

### The role of CNTN1 antibodies and therapeutic approaches

CNTN1-AN antibodies are predominantly of the IgG4 subclass, with IgG3 detectable during acute disease progression.In our case, renal immunofluorescence showed predominant IgG4 and IgG1 anti-CNTN1 deposition along the glomerular basement membrane, though serum antibody subclasses were not tested. Numerous studies have demonstrated that IgG4 subclass CNTN1 antibodies exhibit high affinity for the node of Ranvier and are directly pathogenic [25]. The most pathogenic IgG4 subclass is generated through subclass switching in the sequence of IgG3, IgG1, IgG2, and IgG4 [26]. Norito Kokubun et al. [27] reported antibody subclass switching in four AN patients, noting that IgG1 disappeared as the disease stabilized, suggesting that IgG1 may serve as a potential biomarker for disease activity. Other researchers have proposed that antibody titers, serum neurofilament light chain (sNfL), and serum CNTN1 levels could be useful for monitoring disease activity [3, 28]. IgG3 and IgG1 can activate complement and contribute to acute conduction block and axonal degeneration, correlating with clinical severity during the acute phase [29], while IgG2 shows little C1q binding and IgG4 completely lacks these Fc-mediated effector functions.

IVIG exerts its therapeutic effect by blocking the Fc receptor region of immunoglobulin antibodies, facilitating antibody clearance. However, CNTN1 antibodies, primarily IgG4 subclass, demonstrate poor IVIG response due to limited Fc binding and complement activation. Early-stage patients may show better responses, correlating with antibody profiles. Activated B lymphocytes generate pathogenic anti-CNTN1 antibodies, making rituximab’s B-cell depletion mechanism an effective therapeutic strategy. While early intervention often leads to sustained remission, chronic cases with established axonal degeneration typically demonstrate poorer clinical outcomes [3–5]. Corticosteroids and plasma exchange are also effective treatment modalities.

The standard rituximab regimen (375 mg/m²/week for 4 weeks) is recommended for membranous nephropathy, with approximately 60–70% of patients achieving long-term clinical remission [30]. However, this regimen is costly and associated with a higher incidence of adverse effects. Low-dose rituximab exhibits limited efficacy in PMN, with remission rates below 50%, whereas it demonstrates rapid and sustained therapeutic benefits in anti-CNTN1 nodopathy [31–33]. In our case, considering the predominant neurological involvement with subclinical renal manifestations (proteinuria 4 + but no edema/frothy urine), we initiated low-dose rituximab. This approach resulted in marked neurological improvement but suboptimal renal response. Importantly, this dissociation between neurological and renal improvement occurred despite confirmed complete B-cell depletion (CD19 + count of 0 cells/µL), underscoring that the persistent renal disease was likely driven by pre-existing antibodies and slow tissue repair rather than an ongoing B-cell response. This observation is consistent with existing reports. In accordance with the KDIGO 2021 Glomerular Disease Guidelines [6], we escalated to a dual-target strategy: tacrolimus for rapid podocyte stabilization via calcineurin inhibition [30], complemented by intensified rituximab to eradicate pathogenic B-cell clones. Upon dose intensification with rituximab and the addition of tacrolimus, proteinuria showed further improvement. Scant literature documents the long-term prognosis of CNTN-AN with MN. Notably, earlier MN presentation correlates with better proteinuria remission [8]. Our case aligns with published evidence demonstrating earlier neurological versus renal recovery post-immunotherapy, with MN remission typically requiring high-dose rituximab and/or combination immunosuppression [4, 28, 31, 34].

### CNTN1-AN with concomitant MN

Our patient is an 11-year-old girl presenting with concurrent CNTN1-AN and MN. A study reviewing the clinical features of 35 cases of CNTN1-AN with MN reported up to April 2023 found a mean age of onset of 57.45 ± 2.728 years, with only one case presenting in a patient under 18 years of age [14]. Subsequent studies have reported a rising incidence of CNTN1-AN cases with concurrent MN, with proteinuria identified as a potential biomarker for CNTN1-AN [20]. Therefore, regular monitoring of proteinuria is essential in patients with CNTN1-AN. No statistically significant differences in clinical features or therapeutic outcomes were observed between CNTN1-AN patients with and without MN, except for a higher proportion of acute/subacute onset in the former group. Notably, MN neither exacerbates nor alleviates peripheral neurological symptoms [4].

Anti-CNTN1 Antibody is a potential cause of neuro-renal syndrome. Liu et al. demonstrated that CNTN1 IgG eluted from renal specimens binds to CNTN1 at the node of Ranvier, with IgG4 being the predominant subclass. In contrast, IgG eluted from PLA2R-MN specimens does not exhibit this binding, suggesting that CNTN1 serves as a shared target antigen in both AN and MN [4]. In patients with CNTN1-AN and MN, peripheral nerve symptoms typically precede or coincide with renal manifestations, although rare cases present with MN occurring earlier than AN [8]. The temporal relationship between the onset of these two conditions may depend on whether glomerular damage is primarily mediated by complement activation or results from rapid antibody-mediated disruption of physiological pathways essential for maintaining podocyte foot process integrity. The former mechanism often requires several months to progress to significant proteinuria and nephropathy [35].

The potential mechanisms of CNTN1-mediated renal injury include:①In situ immune complex formation: CNTN1 antibodies bind to CNTN1 expressed on podocyte foot processes, disrupting their normal biological functions.②Exogenous CNTN1 deposition: Circulating CNTN1 implants in the subepithelial space, becoming a target for anti-CNTN1 antibodies.③Circulating immune complex deposition: Immune complexes formed by CNTN1 and anti-CNTN1 antibodies deposit in the subepithelial space. Complement system activation through these pathways induces podocyte injury and glomerular filtration impairment [5]. However, the pathogenesis of CNTN1 antibody-induced neuro-renal syndrome remains incompletely understood. Key unresolved questions include the initial site of CNTN1 exposure to the immune system and the reasons why CNTN1-positive patients develop specific peripheral nerve and/or renal damage.

## Conclusion

CNTN1-AN is rare, with MN comorbidity being relatively common. Regular renal monitoring is crucial in CNTN1-AN patients. Pediatric CNTN1-AN with MN is exceptionally uncommon. We present a pediatric case and characterize this entity to enhance clinical recognition and management. Notably, neurological recovery with low-dose rituximab precedes and surpasses renal improvement. Further studies should investigate the differential efficacy of low-dose rituximab in AN versus MN, optimize treatment protocols (including dosing and combination regimens) for CNTN1-AN with MN.

## Data Availability

All data generated during this study are included in this published article.

## Abbreviations

CNTN1-AN: Anti-contactin-1 antibody-associated autoimmune nodopathy
MN: Membranous nephropathy
CSF: Cerebrospinal fluid
EMG: Electromyography
MRI: Magnetic resonance imaging
CIDP: Chronic inflammatory demyelinating polyradiculoneuropathy
EAN/PNS: European Academy of Neurology/Peripheral Nerve Society
GBS: Guillain-Barré syndrome
IgG: Immunoglobulin G
GBM: Glomerular basement membrane
ANA: Antinuclear antibody
CBA: Cell-based assay
MAG: Myelin-associated glycoprotein
PLA2R: Phospholipase A2 receptor
NELL-1: Neural epidermal growth factor-like 1
THSD7A: Thrombospondin type 1 domain-containing 7 A
DML: Distal motor latency
NCV: Nerve conduction velocity
CMAP: Compound muscle action potential
SCV: Sensory conduction velocity
SNAP: Ssensory nerve action potential
IVIG: Intravenous immunoglobulin
GC: Glucocorticoids
sNfL: Serum neurofilament light chain
